# Evaluation of the Small Changes, Healthy Habits Pilot Program: Its Influence on Healthy Eating and Physical Activity Behaviors of Adults in Louisiana

**DOI:** 10.3390/ejihpe11010019

**Published:** 2021-03-04

**Authors:** Praja Adhikari, Elizabeth Gollub

**Affiliations:** 1 Department of Nutrition, University of North Carolina, Greensboro, NC 27412, USA; p_adhikari@uncg.edu; 2 School of Nutrition and Food Sciences, Louisiana State University Agricultural Center, Baton Rouge, LA 70803, USA

**Keywords:** healthy eating, community-based, nutrition education, lifestyle program

## Abstract

The community-based Small Changes, Healthy Habits (SCHH) program was developed to teach skills and techniques to help adults in Louisiana make and maintain small behavioral changes in their food selection, preparation, and consumption, and in physical activity routines. The content of this four-week program included habit formation and goal setting techniques; physical activity guidance; strategies for a healthier home food environment; a grocery store tour focused on label reading for healthier food selections; basic knife and cooking skills. The program was piloted at ten sites throughout the state. A survey with 14 core items was applied before and after the program to evaluate participant acquisition of skills and behaviors associated with topic areas. A total of 47 participants provided complete data sets. Post-program, these participants reported increased confidence in preparing healthy meals at home (*p* = 0.04); changes in fats (*p* = 0.03) and salt (*p* = 0.01) intake; increased frequency of reading nutrition labels (32%); decreased frequency of meals eaten outside the home (Improvement Index = 0.27); and decreased time/day spent sitting (*p* < 0.05). These short-term results suggest that the SCHH program has potential to positively affect healthy eating and to reduce sedentary behaviors, both of which are fundamental to good health and wellness.

## 1. Introduction

Healthy eating habits and regular physical activity are fundamental to achieve a healthier weight and healthier life. Diet and physical activity patterns are strongly linked to chronic health conditions such as hypertension, heart disease, diabetes, and obesity [1,2]. This assertion is relevant throughout the world, as rates of obesity and non-communicable diseases continue to increase and threaten public health [3]. In the United States, the rate of adult obesity (BMI ≥ 30) has reached 42.4% [4]. Louisiana, with an adult obesity rate of 35.9%, is one of 12 U.S. states with a rate greater than 35% [5]. Louisiana also ranks 46th among the states in sedentary behavior, with an adult physical inactivity rate of 31%, and with less than 20% meeting the Physical Activity Guidelines for Americans [6]. Compared to other U.S. states, Louisiana ranks higher in death rates due to heart disease, stroke and diabetes [7]. The Small Changes, Healthy Habits (SCHH) community-based nutrition education program was developed to help adults throughout Louisiana, establish healthier food and physical activity behaviors as part of their daily routine. Over the long-term, these behaviors could promote maintenance of healthier body weight and reduced risk of chronic diseases. 

The small-changes framework is a behavioral strategy that advocates “conscious small changes in lifestyle behaviors” [8]. It was conceptualized almost two decades ago, as an approach to preventing continuous, gradual weight gain within the population [9]. 

Findings from multiple studies on this subject were compiled in a key report, which concluded that small reductions in energy intake and small increases in physical activities have reduced excessive weight gain [8]. Currently, most Americans are overweight [5] and appear to be more interested in managing their weight by developing lasting, healthier eating habits than by engaging in on-and-off intensive dieting [10]. Yet, many lifestyle-based weight loss interventions have demonstrated only short-term effectiveness [11], with much of the lost weight regained within the first year [12]. A systematic review of weight management strategies and outcomes suggested that it could be more productive for interventions to focus on development of healthy habits in addition to relevant lifestyle change [13]. This research was echoed by a study in which obese individuals described the need for balance with everyday life as important for weight loss and management [14]. 

To be effective, healthy behaviors, whether diet or exercise related, must be consistently and independently maintained. A habit is an automatic behavior developed through repetition over time in a stable context [15]. As an automatic behavior, a habit is less prone to motivational lapse, which is important for long-term behavior maintenance. A medium to strong habit–behavior correlation has been calculated for both healthy eating and physical activity habits, suggesting that habit formation could increase resistance to unhealthy lapses and help sustain these target behaviors beyond a given intervention [16]. Habit formation principles have been applied to behavior change practices with positive outcomes [17,18]. Furthermore, the integration of these principles was found to be acceptable to participants and helpful at modifying and automating behaviors [11]. 

Habits are relevant to maintenance of positive behaviors and a healthier lifestyle [15,17]. The SCHH program integrates habit development techniques with a small change approach to healthier food and physical activity behaviors, to support lasting change. The objective of this evaluation is to determine if the SCHH program can influence positive changes related to healthy eating and physical activity behaviors among participants. 

## 2. Materials and Methods

**Figure 1:**
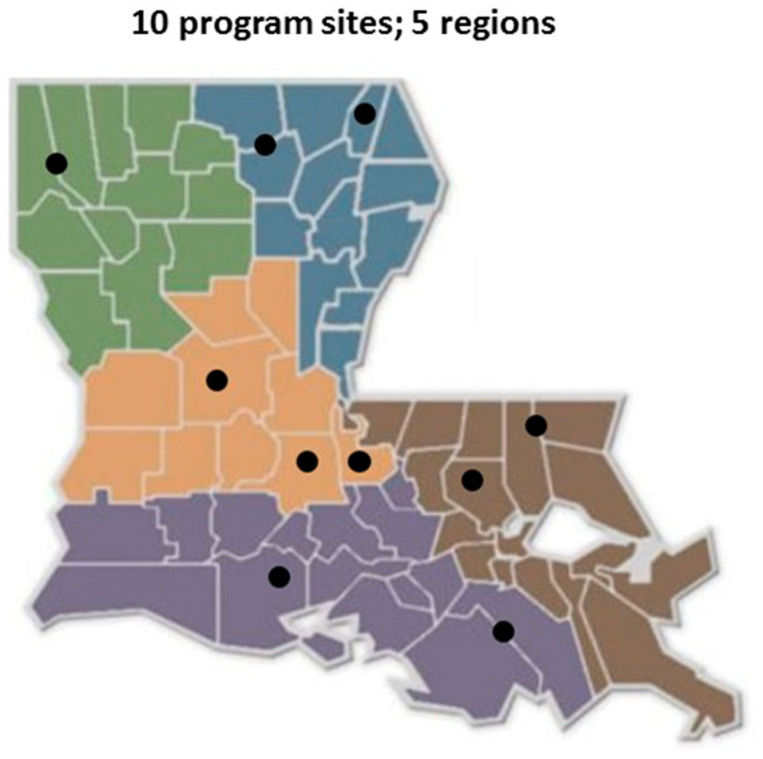
Small Changes, Healthy Habits Implementation Locations in the State of Louisiana.

*Participants:* The SCHH program was available to adults (≥18 years old) in Louisiana. Participants in the evaluation study were those who registered for the program and agreed to participate in the evaluation process, a convenience sample of 47 of the 56 registrants. This pilot program was conducted by 10 nutrition agents, each based in a different community throughout the state (Figure 1). The agents managed recruitment locally, advertising through newspapers and newsletters, with flyers placed in libraries, hospitals, and government agencies, with Facebook posts, and e-mail blasts. There was no charge to enroll in the SCHH pilot program. However, potential participants were asked (though not required) to commit to attending all four sessions of the program and to participate in the evaluation process. Each of 10 nutrition agents recruited 3–15 participants. Basic demographic characteristics of the study participants (sex, race/ethnicity, age, education, employment status, and neighborhood) are presented by category, as a total number and percentage of the study group (Table 1).

*Program description:* The SCHH curriculum conveys information and teaches skills and techniques to help participants make and maintain positive behavioral changes in their food and physical activity routines. The curriculum components are rooted in evidence that food selection, preparation and cooking skills, nutrition information, easy access to healthier foods, physical activity guidance, small goal setting and use of habit formation techniques could influence positive changes in food, eating and physical activity behaviors. The program development team included nutrition, education, and evaluation specialists, and community nutrition practitioners from the Louisiana State University Agricultural Center. This curriculum was designed to be delivered in four sessions, over a four-week period. Each session was to last 90 to 120 min. The day of week and time of day was determined independently by each community nutrition agent; it varied among the 10 program sites. 

Session 1: The first session introduced participants to techniques of habit formation, physical activity strategies, and goal setting processes as applied to small, incremental changes in food and physical activity behaviors, to facilitate weight management [19]. Participants set their own small goal. 

Session 2: The second session focused on creating a healthier home food environment. Participants were taught how to categorize foods in accordance with the traffic light system [20] (green means “go”—anytime; yellow means “slow”—smaller amounts/less frequently; red means “whoa”—once in a while). Participants were also introduced to the concept of choice architecture and its application to the home [21]. SCHH participants practiced strategic placement of healthier foods in easily visible and accessible positions in a mock refrigerator and pantry. 

Session 3: The third session, conducted in a local grocery store, used an experiential learning approach to help participants read and understand the nutrition facts label and additional product label information, to determine healthier food options and make healthier food choices [22]. 

Session 4: The fourth session focused on basic knife and cooking skills, to increase ability and confidence to prepare meals at home, and to encourage more frequent home meal preparation [23,24]. 

Prior to program implementation, the 10 nutrition agents were required to attend a one-day training on delivering the curriculum and facilitating the participant evaluation. For later reference, written instructions on when and how to administer and collect evaluation tools was provided to each agent. To maintain program fidelity across sites, session presentations were scripted and session support activities (e.g., exercise and cooking demonstrations) were prearranged and practiced. 

*Instruments:* The primary evaluation tool was a participant survey containing 14 core items (Table 2). This was used to assess participant’s consumption of key foods and beverages, home food preparation behaviors, food selection and use of nutrition facts labels, and physical activity and sedentary behaviors. The pre-program survey also contained demographic items. Many survey items were adopted from the Behavioral Risk Factor Surveillance System (BRFSS) and the National Health and Nutrition Examination Survey; some were original items intended to assess unique aspects of the SCHH program. A 10-item habit assessment tool was adapted from the Self-Reported Habit Index (SRHI) [25] and the Self-Reported Behavioral Automaticity Index (SRBAI) [26]. This was developed to measure the strength of each participant’s self-selected healthy eating or physical activity goal in terms of the degree of automaticity. 

Procedure and design: The SCHH pilot program was conducted from September to November 2019. The program participant evaluation utilized a pre–post survey design to collect data (self-reported) to capture short-term changes made by participants. Originally, the design also included a 6-month follow-up. However, the COVID-19 pandemic hindered follow-up data collection and value, as access to participants as well as participant routines were significantly altered. Evaluation measures were selected or created to reflect the potential for influence of the core curriculum components on healthy eating and physical activity behaviors among participants. 

The participant survey and habit assessment were both tested prior to implementation, using a cognitive interview process. Revisions to some phrasing and response choices were then made to improve clarity. The pre-program survey was completed immediately before the first session began; the initial habit assessment was completed after the first session because it referred to a small goal determined by the participant during the first session. The post-program survey was completed immediately after the last session. A follow-up habit assessment was to be collected 6 months after the program. 

Healthy eating behavior was measured as frequency over the past 30 days, of consumption of fruits, vegetables, whole grains/cereals, sugar-sweetened beverages, and water (Item 1, Table 2). Participants could choose to report a daily, weekly, or monthly number. Behaviors associated with fats and salt intake were also included (Items 2–3, Table 2). This item was worded to elicit a response of *yes, no* or *not sure*. Confidence in specific knife skills and cooking skills was measured as ratings on a 5-point Likert scale, from 1 (not at all confident) to 5 (completely confident) (Item 7, Table 2). The same scale was applied to measuring confidence in skills related to selecting healthier food options in the grocery store, at restaurants or other prepared food outlets, and in distinguishing between “everyday” and “occasional” foods. (Item 8, Table 2). 

Behaviors related to these skills were measured as frequency of preparing meals at home and of eating meals prepared outside the home (Items 4–5, Table 2). Response choices were provided as small ranges of times/week (e.g., 1–2; 3–4…10 or more). Food selection behavior was measured in terms of how often the participant read and used the nutrition facts label while shopping for food in general and while shopping for foods in specific categories (Items 9–10, Table 2). Response choices were presented as a 5-point Likert scale ranging from always to never. 

The survey contained several items related to physical activity or sedentary behavior. Participants were asked to report the number of days each week they engaged in physical activity and the time (hours and minutes) spent on physical activity on those days (Items 11–12, Table 2). An item comparing the amount of physical activity now to 6 months ago was included for additional comparison during a 6-month follow-up (Item 13, Table 2). Participants were also asked to report the amount of time (hours and minutes) they spent sitting on a typical day (Item 14, Table 2). Common demographic items were included in the pre-program survey. At the end of the program, participants were asked to rate the SCHH program using a 5-point scale, from poor to excellent. They were also asked if they would recommend (yes or no) the program to someone they know. 

*Analysis:* Referring to items in Table 2: Pre-post consumption of specific food categories (Item 1) was compared using paired t-tests; response values were converted to times per day. Pre–post group size of participants watching, reducing, or changing fat or salt intake (Items 2–3) was compared using proportions tests. “Not sure” responses were collapsed into the “no” category for a binary comparison. Items 4, 5 and 6 offered categorical response options related to frequency of food preparation-related behaviors. Here, the improvement index was used to quantify the pre–post changes among the group of participants. Index values are obtained by subtracting the total number of non-favorable changes from the total number of favorable changes, then dividing by the total number of no changes [27]. Changes in frequency of nutrition label reading (Item 9) was also assessed in terms of the improvement index. Item 10, which delt with label reading of specific food categories, was primarily in place for comparison with the (planned) 6-month follow-up. Items 7 and 8, Likert scale confidence ratings of food selection and preparation skills, were analyzed using both the improvement index and the paired t-test. The paired t-test was also used to analyze change in time spent at physical activity or sitting (Items 11, 12, and 14); responses in hours were converted to minutes. Item 13 was in place for comparison with the (planned) 6-month follow-up. All computations were performed using R-statistical software (version 3.6.3) [28].

## 3. Results

All 47 study participants submitted both the pre and post surveys; however, not all participants responded to all survey items. Over the four-week program period, there was no real change among participants in consumption of foods or beverages from any of the targeted categories. However, there was noticeable progress in the sedentary behavior measure. Participants reported a decrease of approximately 1 h/day (61 min) of sitting time. The mean time spent sitting significantly decreased (*p* < 0.01) from 338 min to 277 min per day. The pre to post mean differences in physical activity measured as 0.2 days/week and as 11.1 minuets/day were not significant (Table 3). 

All 47 participants responded to the items that asked if they were watching or reducing their salt or fat consumption or the types of fats they were consuming. Although 40 of these participants experienced no change in how they deal with salt or fat in their diets, seven of these participants made favorable improvements, indicating that they now pay closer attention to dietary salt or fats. As a group, this improvement was significant for fat (*p* = 0.03) and for salt (*p* = 0.01). A similar analysis was also applied to the items on confidence in food preparation skills and food selection skills. No discernible improvements were made by these participants in knife skills or selecting appropriate cooking methods. However, there were significant favorable improvements in confidence to prepare healthy meals at home, with 16 participants improving (*p* = 0.04); to determine healthier prepared food options, with 19 participants improving (*p* = 0.01); to determine healthier options between similar foods, with 23 participants improving (*p* < 0.01); and to distinguish “everyday” from “occasional” foods, with 21 participants improving (*p* = 0.01) (Table 4).

Among these participants, 15 reported a favorable improvement, a reduction, in frequency of consumption of meals/week prepared away from home. There was a complementary favorable improvement among 15 participants who reported increased frequency of home meal preparation (Table 5). There was also positive improvement in the use of nutrition facts labels. For food product purchases in general, 15 participants reported favorable improvements (Table 5). Pre-program, four participants reported that they “Rarely” or “Never” read Nutrition Facts labels; by post-program, all four had shifted to reading labels “Sometimes” or “Most of the time”. This pattern held for all food groups except the juices/teas/sports drinks category (Table 5). Participant responses to the habit assessment yielded no discernible, reliable response pattern.

## 4. Discussion

This SCHH pilot evaluation focused on participant changes in a variety of food and physical activity/sedentary-related factors. During the four-week program period, participants began to watch or reduce their salt and fat intake or change the types of fats they are using. Participants learned to use food labels or product information to select healthier foods in grocery stores and in prepared food outlets. Participants also increased their confidence in food selection and preparation skills, which coincided with an increase in home meal preparation. Participants reduced daily sitting time, though this did not translate to significant increases in physical activity. 

These results are similar to a recent six-week healthy lifestyle program in Washington State, targeting obese Hispanic women [29]. After a series of community workshops focused on nutrition and physical activity, a group of 49 women demonstrated an increase in nutrition label literacy and in physical activity, and a decrease in consumption of food eaten in restaurants. These indicators of healthier lifestyle are important because of the potential to impact health. 

Cooking skills are another indicator. Individuals who lack cooking skills are more likely to eat away-from-home or purchase convenience foods on a regular basis; foods obtained away from home generally have higher sodium, kilocalories, saturated fat and cholesterol [30]. In a community-based study involving middle-age and older adults learning nutrition and cooking skills, researchers observed a significant positive association between cooking knowledge and cooking confidence, and between cooking confidence and dietary habits [31]. Approximately 89% of the SCHH pilot participants were middle-age to older adults. There was no improvement in cooking skills confidence among SCHH participants; however, there was a significant improvement in their confidence to prepare healthy meals at home. It is possible, especially given their age, that these SCHH participants were already confident in their cooking skills.

The home food environment, the availability and accessibility of foods, influences food selection and consumption [32,33]. As a concept, this is referred to as choice architecture, which assumes that, at the point of decision making, the easiest choice is the more probable one; food placement can influence selection [21]. This approach is being encouraged in public school cafeterias [34]; in conjunction with the Food Traffic Light System, it has had success in business, improving sales of healthier foods [35]. The SCHH program presented approaches to application of this concept to the home (e.g., repositioning foods in the refrigerator, in the pantry, on countertops). Although program participants increased their confidence in determining healthier food options, there was no real change in their consumption of foods in the healthy eating categories of interest. 

The SCHH program focused on how to read and use the food label, which is recognized as a primary tool for assisting the public in making healthier food choices [22]. Understanding the label enables people to make informed food choices, especially in reference to sodium, fat, fiber, whole grain, and added sugars. SCHH participants improved their use of food labels, and their confidence to determine healthier food options. Yet, they did not change consumption patterns. Participant data hinted at the intended change in consumption of fruits, dark green vegetables, drinking water and regular soda, which might require longer than one-month to establish. It is also possible that the food consumption tool could not detect change in this group size. An alternative survey item will be used going forward. Participant intake of sugar-sweetened beverages did not change at all, suggesting that additional emphasis on non-sugar-sweetened beverage alternatives be added to the curriculum. A recent study examining strategies for promotion of healthy eating found that sugary beverages were among the most difficult “foods” to control, and that gradual (small) dietary changes were among the strategies found to be most effective [36]. 

Physical Activity plays a critical and complementary role in health and wellness. For this reason, a recommendation to meet the Physical Activity Guidelines for Americans is included in the Dietary Guidelines for Americans 2020–2025 [37]. A sedentary lifestyle contributes to obesity, cardiovascular diseases, type 2 diabetes, and some cancers, whereas physical activity of any kind or duration helps diminish risk to these diseases, improves longevity, and promotes a healthier life [38,39]. Over the SCHH program period, participants significantly reduced, by 61 min/day, the time they spent sitting. As part of the SCHH program, participants were asked to create a small physical activity goal. These physical activity goals might have contributed to this reduction in sedentary behavior. Time spent being physically active did not significantly increase; yet, the additional 11 min/day is beneficial, and should not be discounted. 

Habit formation techniques to help transform small conscious behavioral changes to automatic behaviors are an integral component of this program. The evaluation attempted to measure habit strength progress. However, the habit assessment tool, found to be acceptable and functional during preliminary testing, did not work with the pilot evaluation. Post-program participant interviews (not described here) indicated that the habit assessment was confusing and complex, preventing participants from responding in a meaningful way. Alternative formats were discussed. A simplified version will be tested during the next program implementation. 

The evaluation was conducted with a relatively small and homogeneous group of participants, not necessarily representative of the state. These participants gave high ratings to the program, but they also suggested program adjustments. The participant survey and the habit assessment were formatted for a traditional face-to-face experience. However, an electronic version of the program and the participant evaluation will now be developed to increase reach and facilitate both short and longer-term follow-up, even during times of social distancing. As modified, the SCHH program will be further tested with a larger, more diverse audience. 

## 5. Conclusions

The Small Changes Healthy Habits pilot program was evaluated for effectiveness at influencing healthy eating and physical activity behaviors among participants. This evaluation utilized participant self-reports over a four-week period; too short to see large and/or significant changes in behaviors—but enough to see significant progress in many important areas. These short-term results suggest that the SCHH program has the potential to positively affect healthy eating and reduce sedentary behaviors, both of which are fundamental to good health and wellness. The improvements in healthy eating and physical activity-related behaviors presented here are precisely the type of small changes that could have a large health impact over a lifetime.

## Figures and Tables

**Table 1:** Basic Demographic Characteristics of Small Changes, Healthy Habits Evaluation Study Participants.

Characteristic	Numbers	Percentage (%)
Sex (n = 47)		
Male	8	17
Female	39	83
Age (years) (n = 46)		
18–34	4	9
35–50	1	2
51–64	21	46
65+	20	43
Ethnicity (n = 46)		
White	38	83
Black or African American	6	13
Asian	1	2
Prefer not to say	1	2
Education Level Completed (n = 47)		
High School	10	21
Some college or vocational school	9	19
2-year degree	3	6
4-year degree	12	26
Some graduate school	11	24
Prefer not to say	2	4
Neighborhood (n = 46)		
Rural	41	89
Urban	5	11
Employment status (n = 46) *		
Work part-time	5	11
Work full-time	15	33
Work from home	5	11
Work outside the home	21	46
Retired	21	46
Prefer not to say	2	4

* Participants could select more than one category.

**Table 2:** Core Items of the Small Changes, Healthy Habits Participant Survey.

Item No.	Participant Survey Items *
1	During the past 30 days, how many times did you eat the following types of food?
	Fruits Dark green vegetables Orange or red colored vegetables Whole grain breads Regular soda Sugar sweetened fruit drinks, sweet tea, sports or energy drinks Water
2	Are you currently watching or reducing your fat intake or changing the type of fat you use?
3	Are you currently watching or reducing your sodium or salt intake?
4	During the past week, how many meals did you eat that were prepared away from home?
5	In a typical week, how many times do you prepare a main meal from whole foods that need to be washed, cut, seasoned, and/or cooked?
6	On days that you go to work, school, take road trips or are just out and about/away from home, do you prepare and pack your own meals or snacks?
7	Using a scale of 1 to 5, how confident are you in your ability to…
	Select the right knife for the job? Slice, dice, mince, chop and peel vegetables? Select appropriate cooking methods (e.g., roasting, sautéing, grilling, stewing, baking) for your food? Prepare healthy meals at home?
8	Using a scale of 1 to 5, how confident are you in your ability to…
	Determine which foods are the healthier options when selecting foods from restaurants, food stands, fast food outlets, vending machines, etc.? Determine which item is the healthier option when choosing between similar food items at the grocery store? Distinguish between “every day” foods and “occasional” foods?
9	How often do you use the Nutrition Facts label when deciding to buy a food product?
10	How often do you look for nutrition information on the Nutrition Facts label when you buy each of the following types of foods?
	Snacks (chips, crackers, popcorn, cookies, candies) Breakfast cereals Salad dressings, fats, oils Raw meat, fish, seafood, poultry Processed meat, fish seafood, poultry Breads, tortillas Milk, yogurt, cheese Juices, teas, sports drinks
11	In a typical week, on how many days do you do any type of physical activity that causes an increase in breathing or heart rate?
12	For the number of days reported above, on average, how much time per day do you generally spend doing these physical activities?
13	Compared to 6 months ago, would you say that you are now participating in less, about the same, or more physical activity?
14	On a typical day, how much time do you usually spend sitting?

* This table lists the core survey items; the actual survey included a description, example, or context for these core items.

**Table 3:** Pre-Post Comparisons of Food/Beverage Consumption and of Time spent at Physical Activity/Sedentary Behavior.

Food/Beverage Consumption (Times/Day)	n	Pre-Mean	SD ^1^	Post-Mean	SD^1^	*p*-Value ^2^
Fruits	45	1.55	3.71	1.74	3.73	0.26
Dark-Green vegetables	46	0.95	1.02	1.03	1.02	0.46
Orange-red colored vegetables	46	0.73	0.98	0.67	0.93	0.97
Whole grain breads	44	0.98	1.31	0.90	1.34	0.95
Whole grains or cereal	45	0.82	1.23	0.63	0.69	0.45
Regular (not diet) soda	46	0.14	0.35	0.12	0.35	0.67
Sugar sweetened beverages (not soda)	46	0.32	0.94	0.32	0.97	0.33
Water	39	4.38	2.60	4.58	4.88	0.20
Physical Activity/Sedentary Behavior						
Days/week of physical activity	45	3.76	1.85	3.93	1.73	0.33
Minutes/day of physical activity	42	62.66	67.28	73.76	78.78	0.14
Minutes/day of sitting	45	338	132	277	167	<0.01

^1^ SD = standard deviation. ^2^ *p* < 0.05; paired t-test.

**Table 4:** Pre-Post Changes in Fat and Salt Behaviors, and Confidence in Food Selection and Preparation.

Survey Item	n	FI ^1^	NFI ^2^	NC ^3^	II ^4^	*p*-value *
Currently watching, reducing, or changing type of fat	47	7	0	40	0.18	0.03 *
Currently watching or reducing salt	47	7	0	40	0.18	0.01 *
***Confidence in ability to:***						
Select right knife for task	46	16	12	18	0.22	0.66
Slice, chop, peel veggies	44	13	10	21	0.15	0.72
Select appropriate cooking methods	45	16	8	21	0.38	0.08
Prepare healthy meals at home	45	16	5	24	0.45	0.04*
Determine healthier prepared food option	46	19	7	20	0.60	0.01*
Determine healthier option between similar foods	46	23	5	18	1.00	<0.01*
Distinguish “everyday” from “occasional” foods	46	21	10	15	0.73	0.01*

^1^ FI = Favorable Improvement. ^2^ NFI = Non-Favorable Improvement. ^3^ NC = No Changes. ^4^ II = Improvement Index (favorable change—non favorable change/no change). * *p* < 0.05.

**Table 5:** Improvement Index Factors for Meal Preparation and Use of Nutrition Facts Labels.

Survey Item	n	FI ^1^	NFI ^2^	NC ^3^	II ^4^
Meals at home or outside					
Meals prepared away from home	47	15	9	22	0.27
Meals prepared at home (from whole foods)	47	15	11	21	0.19
Nutrition Facts Label reading behavior					
Use nutrition fact label	45	15	5	25	0.40
Read labels for snack item	45	14	9	20	0.25
Read labels for breakfast item	45	11	5	27	0.22
Read labels for salad dressings	45	14	8	21	0.28
Read labels for meat	45	17	10	17	0.41
Read labels for processed meat	45	16	12	15	0.26
Read labels for bread	45	15	5	23	0.43
Read labels for dairy product	45	17	12	15	0.33
Read labels for juices/teas/sports drinks	45	10	11	21	-0.04

^1^ FI = Favorable Improvement. ^2^ NFI = Non-Favorable Improvement. ^3^ NC = No Changes. ^4^ II = Improvement Index (favorable change—non favorable change/no change).
